# Influence of RNA extraction methods and library selection schemes on RNA-seq data

**DOI:** 10.1186/1471-2164-15-675

**Published:** 2014-08-11

**Authors:** Marc Sultan, Vyacheslav Amstislavskiy, Thomas Risch, Moritz Schuette, Simon Dökel, Meryem Ralser, Daniela Balzereit, Hans Lehrach, Marie-Laure Yaspo

**Affiliations:** Max Planck Institute for Molecular Genetics, Ihnestr. 63-73, Berlin, 14195 Germany; Novartis Institutes for Biomedical Research, Biomarker Development, Fabrikstr. 10, Basel, Switzerland

**Keywords:** RNA-Seq, RNA extraction, rRNA depletion, poly(A)+ selection, Intronic reads

## Abstract

**Background:**

Gene expression analysis by RNA sequencing is now widely used in a number of applications surveying the whole transcriptomes of cells and tissues. The recent introduction of ribosomal RNA depletion protocols, such as RiboZero, has extended the view of the polyadenylated transcriptome to the poly(A)- fraction of the RNA. However, substantial amounts of intronic transcriptional activity has been reported in RiboZero protocols, raising issues regarding their potential nuclear origin and the impact on the actual sequence depth in exonic regions.

**Results:**

Using HEK293 human cells as source material, we assessed here the impact of the two commonly used RNA extraction methods and of the library construction protocols (rRNA depletion versus mRNA) on 1) the relative abundance of intronic reads and 2) on the estimation of gene expression values. We benchmarked the rRNA depletion-based sequencing with a specific analysis of the cytoplasmic and nuclear transcriptome fractions, suggesting that the large majority of the intronic reads correspond to unprocessed nuclear transcripts rather than to independent transcriptional units. We show that Qiagen or TRIzol extraction methods retain differentially nuclear RNA species, and that consequently, rRNA depletion-based RNA sequencing protocols are particularly sensitive to the extraction methods.

**Conclusions:**

We could show that the combination of Trizol-based RNA extraction with rRNA depletion sequencing protocols led to the largest fraction of intronic reads, after the sequencing of the nuclear transcriptome. We discuss here the impact of the various strategies on gene expression and alternative splicing estimation measures. Further, we propose guidelines and a double selection strategy for minimizing the expression biases, without loss of information.

**Electronic supplementary material:**

The online version of this article (doi:10.1186/1471-2164-15-675) contains supplementary material, which is available to authorized users.

## Background

Next generation sequencing (NGS) has become the gold standard for in depth transcriptome analysis, since RNA sequencing (RNA-seq) provides a high dynamic range and a virtually unbiased view of the transcriptome landscape [1, 2] although, several studies have pointed out limitations, which might reflect variations in experimental procedures [3–5]. The most commonly used methods for extracting total RNA from cells or tissues are the phenol-Chloroform based (e.g. TRIZol) and the silica-gel based column procedures (e.g. Qiagen). The RNA-seq libraries generated prior to the sequencing are based either on selecting poly(A)+ messenger RNAs, or on depleting total RNA of highly abundant ribosomal RNAs. The rRNA depletion protocols offer an attractive option, facilitating the simultaneous characterization of polyadenylated and non-polyadenylated RNAs, including non-coding RNAs, while requiring minimal amounts of starting RNA material [6, 7]. Comparisons of mRNA expression values between poly(A)+ -selected and rRNA-depleted libraries highlighted discrepancies, raising issues in the accurate estimation of gene expression levels [4, 6]. It has been shown that RNA-seq data originating from rRNA-depleted procedures are characterized by a significant number of reads mapping to non-coding regions, which were for a large part localized within introns [8–11]. However, the relative abundance of intronic transcripts as compared to the expression level of coding exons was reportedly very variable between samples and studies, and these differences have been up to now mostly attributed to the biological contexts [8, 10], although one cannot rule out the influence of the experimental procedures. One essential issue for interpreting these differences is to be able to distinguish the intronic reads corresponding to unspliced immature precursor mRNA (hnRNA) from those defining distinct transcriptional units, such as long non-coding RNAs [8, 9, 12]. This is particularly relevant since the concomitant presence of mature and immature transcripts will have a direct impact on downstream analysis of gene expression profiles.

Here, using HEK293 human cells as source material, we set out to assess the influence of the RNA extraction methods (TRIzol versus silica gel) and of the library construction protocols (rRNA depletion versus poly(A)+ selection) on 1) the relative abundance of intronic reads and 2) on the estimation of gene expression values. Further, in order to benchmark this information, we sequenced both the cytoplasmic and nuclear RNA fractions of HEK293 cells to investigate the origin of the intronic reads observed in rRNA-depleted RNA-seq procedures. Based on the data generated, we discuss the respective performances of the different protocols in detecting the non-polyadenylated and non-coding fractions of the transcriptome, and their impact for analyzing the transcriptome landscape in general.

## Results

### Differences in intronic read abundance are protocol dependent

**Figure 1:**
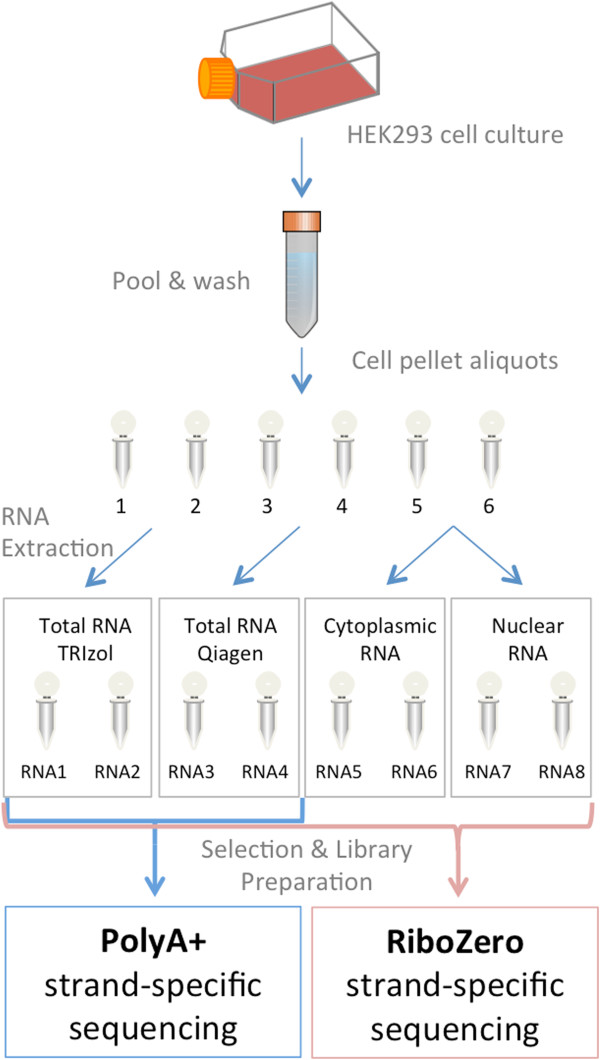
**Experimental workflow.** HEK 293 T cells were used to assess the influence of RNA extraction protocols in the RNA-seq data after sequencing with poly(A)+ or RiboZero procedure. The HEK293 nuclear and cytoplasmic RNA fractions provided a benchmark for the Ribozero sequencing.

We carried out a comparative sequence analysis of the total, nuclear, poly(A)+ and cytoplasmic RNA fractions of HEK293 cells, extracted by either organic or non-organic methods, respectively with the purpose of investigating the influence of RNA extraction and library preparation protocols on RNA-seq data analysis (Figure 1). All libraries were done using strand-specific protocols and sequenced on a HiSeq2500 instrument.

Here, we used the RNeasy preparation method (Qiagen), the phenol-chloroform based TRIzol procedure, and we separated the nuclear and the cytoplasmic fraction using the non-organic extraction kit PARIS (Life Technologies). All RNA extractions were performed in duplicates. We detected nuclear unspliced RNAs (bioanalyzer peak >4000 nt) in TRIzol RNA, Qiagen RNA and nuclear RNA, whereas none could be seen in the cytoplasmic RNA fractions (Additional file 1: Figure S1). A clear difference was also seen for small RNAs (<200 nucleotides), which were found more abundant in TRIzol-extracted RNAs, as compared to all other methods, in line with the fact that the silica column-based method do not retain smaller RNAs (<70 nucleotides according to the manufacturer).

**Figure 2:**
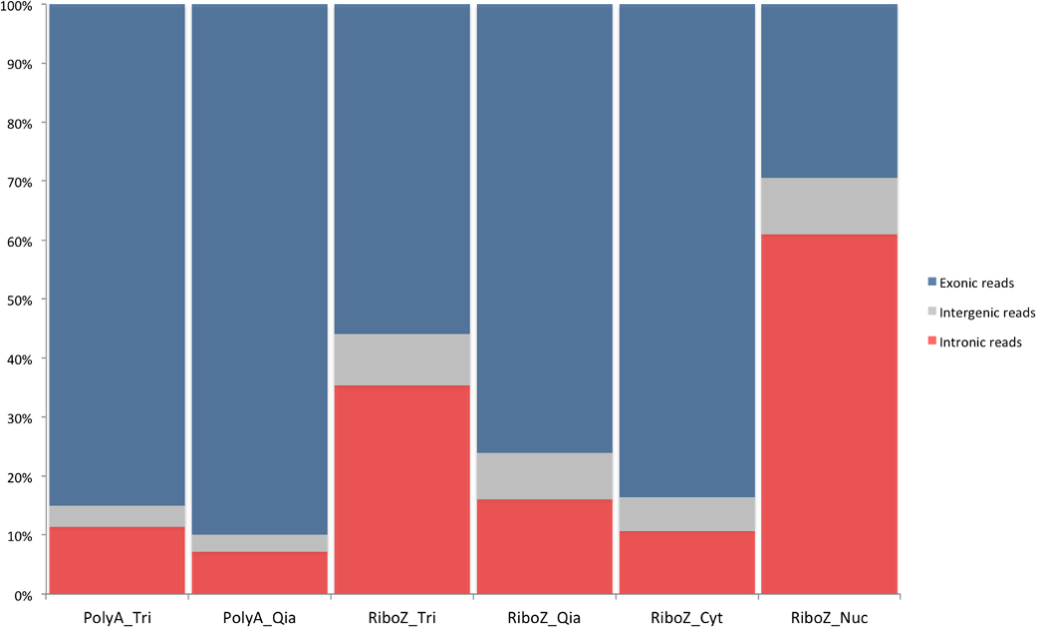
**Distribution of exonic, intronic and intergenic reads in the six combinations of experiments.** The barplot shows for each sequencing group the respective fraction (%) of the sum of aligned read of each replicate. *Tri*: TRIzol RNA; *Qia*: Qiagen RNA; *RiboZ* = RiboZero; *Par* = PARIS RNA extraction kit (Ambion); *cyt*: cytoplasm RNA; *nuc*: nuclear RNA.

For all tested protocols (Figure 1), we sequenced on average 111 millions reads of 51 bases per sample, out of which ~94% could be mapped to the human reference genome (Table 1). Only a tiny fraction of reads corresponding to ribosomal RNAs (0,3%) was seen in the poly(A)+ RNA-seq but almost none in the RiboZero method, assessing the selection efficiency. However, we observed substantial differences in the exonic and intronic read distribution, depending on the RNA extraction method and RNA-seq selection procedure (Figure 2, Table 1).

**Table 1:** **Mapping statistics**

Group	Sample	All reads	Mapped reads	Pairs	Mapped, MAPQ > 1	Mapped on exons (%)	Mapped on introns (%)	Mapped on introns (%) mapped to non annotated regions (%)	Mapped on rRNA (%)	Mapped on mitochondrial DNA (%)	Unique starting position (%)
PolyA Trizol	Tot_RNA1	101028616	93699257	78996022	83038355	86,7	10,7	2,6	0,31	4,80	32,4
	Tot_RNA2	103480788	96072826	81214720	84983881						
PolyA Qiagen	Tot_RNA3	100766522	100766522	76780490	82231228	91,3	6,6	2,1	0,30	4,58	28,5
	Tot_RNA4	109645220	109645220	83720316	89300384						
RiboZ Trizol	Tot_RNA1	109221096	103587793	92825224	85242138	60,6	35,1	4,3	0,04	0,39	51,5
	Tot_RNA2	113936740	107997806	96763362	90980604						
RiboZ Qiagen	Tot_RNA3	118950948	118950948	110394536	91193892	81,6	15,6	2,8	0,02	0,28	35,1
	Tot_RNA4	112056778	112056778	88450950	86755352						
RiboZ cytoplasmic	Cyt_RNA5	111517838	102989353	86770814	87248867	87,1	10,3	2,6	0,03	0,56	31,3
	Cyt_RNA6	102998772	94630661	79109400	87248867						
RiboZ nuclear	Nuc_RNA7	126297526	122854406	116663678	115579529	31,1	60,8	8,2	0,01	0,05	65,2
	Nuc_RNA8	127905406	124011269	117109650	116428981						

The numbers of mapped sequence reads are given as absolute numbers. Percentages are calculated according to the total number of reliable reads (MAPQ > 1). The number of unique starting positions is a measure of the library complexity.

As expected, we observed the highest fraction of exonic reads for the poly(A)+ selected libraries, without significant difference in the exonic coverage between the procedures using Qiagen or TRIzol RNA extractions (91% and 87% reads mapping to exons, respectively). In contrast, the data obtained with RiboZero RNA-seq were highly sensitive to the RNA extraction methodology. In fact, the RiboZero procedure only generated data comparable to that of the poly(A)+ RNA-seq when using cytoplasmic-fractionated RNAs (87% of exonic and 10% intronic reads), whereas nuclear-fractionated RNA processed with RiboZero led to 31% of exonic and 61% intronic sequences reads (Figure 2). Results were more mitigated with the more common RNA extraction methods. RiboZero RNA-seq showed twice as many intronic reads for TRIzol-extracted than for Qiagen-extracted RNA (35% and 16%, respectively) (Figure 2 and Table 1). Most of those intronic reads were in the same orientation as their corresponding mRNA (82% and 70% of the intronic reads for the TRIzol and Qiagen RiboZero RNA-seq respectively), strongly suggesting that they were associated with the corresponding immature hnRNAs. Taken together, these data suggest that the combination of TRIzol RNA extraction with RiboZero RNA-seq protocol tend to produce a significant fraction of intronic sequence reads, which are likely to have a nuclear origin, pointing out to partially or unprocessed RNAs species (hnRNA). The majority of intronic reads do not belong to antisense transcripts, although we cannot excluded the presence of functionally independent RNAs that are collinear with the mRNA of the host gene [12]. Consistent with previous results [9, 12] the bulk of intronic reads represented the majority of the non-exonic RNA sequences in our dataset, with only a small fraction being intergenic (Figure 2). However, the RiboZero method detected slightly more transcriptional activity in non-annotated regions (2.8-4.3% of the reads) than the poly(A)+ RNA-seq procedure (2.1-2.6%) (Table 1) pointing out to yet uncharacterized non-polyadenylated RNA species. In total, we found 5.7 Mb of non-annotated sequences potentially transcribed in the RiboZero method with a minimum coverage of 2 reads. In all cases, the coverage in non-annotated regions was slightly higher in TRIzol RNA over Qiagen RNA (Table 1). Besides, we noted that transcripts encoded by the mitochondrial genome were better covered with the poly(A)+ RNA-seq approach (Table 1), consistent with the fact that human mitochondrial transcripts possess stable 3’-end poly(A) tails and are thus enriched through this selection method [13, 14].

### Detection and expression of protein coding genes

**Figure 3:**
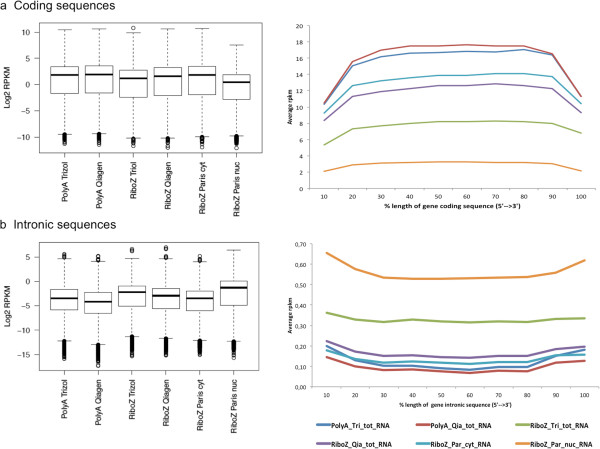
**Expression and coverage of exonic and intronic reads.** Boxplots (left panels) representing the mean exonic **(a)** and intronic **(b)** expression values of the two replicates of each experimental group. The corresponding coverage is shown on the right panels. Merged exons (a) and intron (b) length for each detected gene were divided in 10 bins, and the average expression value (rpkm) was calculated for each bin.

The qualitative variations observed between protocols, raised issues regarding the estimation of expression levels of coding genes. We calculated the expression values in reads per kilobase per million (rpkm) [1] for each annotated gene in Ensembl (Methods). The Pearson correlation of gene expressions between two replicates of each experimental group was high (r ≥ 0.99; Additional file 1: Figure S2), confirming the known high technical reproducibility of NGS [2]. From 20,234 annotated protein-coding genes (Ensembl v.70), 62% were found expressed (rpkm ≥ 0.5) in the nucleus and 60% in the cytoplasm (Table 2) of HEK293 cells, respectively, and 93% of the genes expressed in the nuclear compartment were also detected in the cytoplasm (Additional file 2: Table S1). The 465 genes found only in the cytoplasmic fraction were in majority low expressed genes (average 1.2 rpkm), and could be detected in the nuclear fraction albeit below the detection threshold (average 0.3 rpkm). Overall, a similar number of protein coding genes was detected by all methods (Table 2). The global distribution of coding sequence expression (rpkm values) was similar across methods, albeit slightly lower in RiboZero-TRIzol RNA and nuclear fraction whereas as expected, the rpkm values of intronic sequences showed the reverted trend (Figure 3a, left panel). The read sequence coverage along coding sequences measuring messenger RNAs (in rpkm) was the highest in the poly(A)+ method and the lowest for the nuclear RNA fraction (Figure 3a). In contrast, the RiboZero total RNAs showed clear differences in coverage depth depending on the extraction method, following the trend described above where Qiagen-extracted total RNA was more similar to cytoplasmic RNA results, while TRIzol-extracted RNA was markedly lower, and was the second lowest after the nuclear RNA.

**Table 2:** **Transcript coverage**

	Categories	PolyA Tot RNA Trizol	PolyA Tot RNA Qiagen	RiboZeroTot RNA Trizol	RiboZeroTot RNA Qiagen	RiboZero Cytopl. RNA	RiboZero Nuclear RNA
Protein coding genes	Genes with rpkm ≥ 0.5	12469	12381	12164	12164	12119	12498
	Average genes coverage	67%	65%	69%	68%	65%	71%
	Genes not covered	2259	2447	2016	2085	2316	1778
	Genes ≥50% covered	14345	14144	14540	14540	14038	14978
	Genes 100% covered	1677	1468	2287	2072	1370	2892
	Genes with only 1 read	263	1468	278	292	287	261
	Genes with ≥5 reads	16926	1468	17164	17067	16844	17464
	Genes with ≥50 reads	14768	14667	14812	14836	14597	15190
	Genes with ≥100 reads	14111	14049	13997	14071	13888	14309
Long non-coding RNAs	processed transcript	423	418	452	417	404	532
	lincRNA	596	543	625	546	521	927
	antisense	914	850	748	748	722	911
	sense intronic	97	68	269	269115	87	499
	sense overlapping	58	44	57	42	44	87
	3 prime overlapping ncrna	20	20	19	19	20	22
	non coding	4	3	4	4	4	5

The upper panel lists the number of detected and covered protein coding genes in each category and experimental condition. The panel below lists for each experimental condition the number of detected long non-coding RNA subtypes (rpkm > =0.5).

The rpkm expression values for the cumulative intronic sequences of each gene were the highest for TRIZol-RiboZero RNA-seq, after the nuclear RNAs (Figure 3b). Within genes, intronic and exonic expression levels were highly correlated in RiboZero TRIzol RNA (Pearson coefficient r = 0.83) and RiboZero Qiagen RNA data (Pearson coefficient r = 0.77), in agreement with previously published results suggesting that most of the intronic signal is originating from pre-mRNA or splicing by-products and does not represent stand-alone functional RNAs [8, 10]. This view has been challenged by the notion that such signals are part of the pervasive transcription of the genome and not necessarily associated to known genes [9, 12, 15].

Comparative analysis of TRIzol and Qiagen RNAs sequenced by either poly(A)+ or RiboZero protocols, revealed clear differences in the coding sequences sequencing depth, which was inversely proportional to that of intronic sequences, an observation, which can be logically explained by the sampling factor.

### Consequences for gene expression analysis

**Figure 4:**
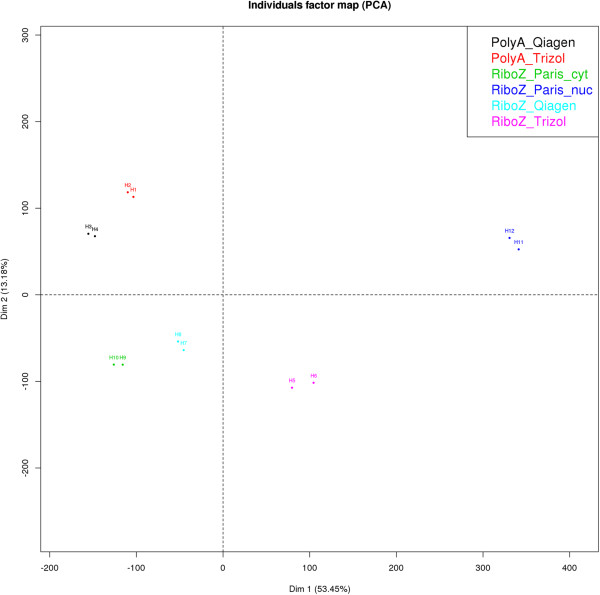
**Principal Component Analysis (PCA) of the six experiments.** All gene expression values where used to perform the PCA (CRAN Package FactoMineR, Version 1.24).

We investigated how those differences between protocols impacted gene expression values. We applied principal component analysis (PCA) and pairwise correlations on the various dataset obtained for HEK293 (Figure 4, Additional file 1: Figure S3). Data generated from the two total RNA extraction methods (Qiagen vs TRIzol) were highly similar if they were sequenced with the same protocol, either poly(A)+ (r > 0.99) or RiboZero. (r > 0.98) (Additional file 1: Figure S3). However, in line with the data mentioned above, the RiboZero-Qiagen RNA combination generated a profile similar to that of cytoplasmic RNA (r > 0.99) (Figure 4, Additional file 1: Figure S3). The largest differences in gene expression profiles were observed between poly(A)+ and RiboZero RNA-seq, with Pearson’s correlation coefficients ranged from 0.93 to 0.96 [6, 16]. The nuclear RNA expression pattern was the most different from all, harboring the lowest pairwise correlations (Figure 4, Additional file 1: Figure S3).

We measured the variation in gene expression values seen in the series of HEK RNA-seq experiments in a comparative analysis using the NOISeq differential expression algorithm [17] (see Methods). Genes with a probability higher than 80% (see methods) were considered as differentially expressed between protocols (Additional file 3: Table S2). Table 3 shows the number of differentially expressed coding transcripts between the different methods and shows that within a given RNA-seq protocol, data were globally comparable, albeit with some differences (Table 3). Looking at the influence of RNA extraction methods, we detected only 21 genes whose expression values were significantly different between Qiagen and TRIzol RNAs using the poly(A)+ RNA-seq and 79 genes in the RiboZero RNA-seq. Of those, nearly all genes displayed higher expression levels for TriZol versus Qiagen RNA and were expressed at low to medium levels (<10 rpkm) (Additional file 3: Table S2).

**Table 3:** **Differential expression**

	Down	Up
PolyA Qiagen vs PolyA Trizol	17	4
PolyA Qiagen vs RiboZ Qiagen	141	684
PolyA Trizol vs RiboZ Trizol	344	723
RiboZ Qiagen vs RiboZ Trizol	77	2
PolyA Qiagen vs RiboZ Trizol	313	729
PolyA Trizol vs RiboZ Qiagen	158	681
RiboZ cyt vs RiboZ nuc	2295	2671

The table lists the number of protein coding genes detected as differentially expressed by the NOISeq algorithm across the different protocols.

In contrast, the gene expression differences were more drastic between poly(A)+ and RiboZero RNA-seq, (Table 3). In total, 423 unique protein coding genes were seen significantly more expressed in RiboZero RNA-seq than in poly(A)+ RNA-seq, among which 46 genes belonged to the replication-dependent histone cluster known to be non-polyadenylated genes.

To detect specifically the non-polyadenylated protein coding genes in our dataset, we applied arbitrary and restrictive filters, which included the average difference in rpkm between RiboZero and poly(A)+ (>5 rpkm) and the fold change (>2). In total, 74 protein coding genes met these criteria, which included 43 out of the 46 replication-dependant histone cluster genes that were identified as differentially expressed above (Additional file 2: Table S1). Over two third of the non-polyadenylated genes were still detected in poly(A)+ samples, albeit at dramatically lower levels than in the RiboZero data. This includes 28 of the histone cluster genes, corroborating studies showing that polyadenylated histone transcripts from replication-dependent histone genes can be produced due to the loss of correct 3′ end processing [18–20]. The differences between cytoplasmic and total RNA were smaller than between nuclear and total RNA. Almost all protein coding genes were seen expressed at higher levels in total RNA given that total RNA contains the poly(A)+, the poly(A)- as well as bimorphic classes of RNAs [5, 7].

**Figure 5:**
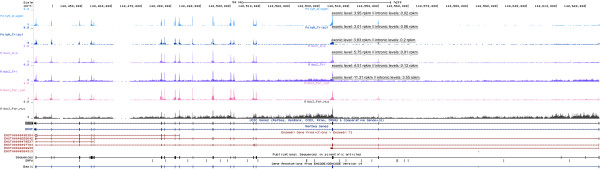
**BRAF gene coverage.** Snap shot (UCSC browser) representing the normalized read coverage for each method and the protocol dependence of the intronic reads. From top to bottom the experimental groups are the following: Poly (A) Qiagen total RNA, Poly (A) TRIzol total RNA, RiboZero Qiagen total RNA, RiboZero TRIzol total RNA, RiboZero cytoplasmic RNA, RiboZero nuclear RNA.

On the other hand, 1,075 unique genes were expressed at higher levels in poly(A)+ RNA-seq than in RiboZero RNA-seq, of which more than half showed exonic values greater than 10 rpkm (Additional file 3: Table S2). For these genes, the corresponding intronic expression levels were conversely significantly higher in TRIzol RNA samples and/or in RiboZero RNA-seq data, as compared to Qiagen RNA and/or poly(A)+ -seq (Additional file 1: Figure S4), suggesting the presence of varying amounts of pre-mRNA species. This is illustrated by the BRAF gene found more expressed in RiboZero TRIzol RNA versus poly(A)+ TRIzol RNA (1,9 fold) and in RiboZero nuclear RNA versus cytoplasmic RNA (2,5 fold) (Figure 5). Indeed the intronic expression levels of BRAF were the highest in RiboZero TRIzol RNA (0,91 rpkm) and RiboZero nuclear RNA (3,55 rpkm), in contrast to e.g. cytoplasmic RNA (0.12 rpkm) (Figure 5). However, this did not fully explain why most of the genes with varying expression were detected at higher levels in poly(A)+ RNA sequencing. A closer examination, showed significant differences in the size distribution of the intronic and exonic sequences between over- and under- expressed genes in poly(A)+ RNA-seq (Additional file 1: Figure S5). Protein coding genes with higher expression in poly(A)+ RNA-seq had a longer intronic (median = 73,4 kb; p_Kolmogorov-Smirnov_ < 2.2e-16) and exonic (median = 5.8 kb, p_Kolmogorov-Smirnov_ = 2.4e-14) sequence length than those genes with higher expression in RiboZero RNA-seq (median_intron_ = 16,1 kb; median_exon_ = 3,8 kb). These observations corroborate the reads sampling factor contribution to the apparent different expression. For any given gene whose pre-mRNAs are sequenced along with the matured RNAs, the overall expression of the coding parts will appear lower if the protocol favors the presence of nuclear RNAs. However, we did not see a correlation between the size of the coding sequences and the number of genes seen with varying levels of expression between protocols, in contrast to previously reported results [4] (Additional file 1: Figure S6).

### Detection of long non-coding RNAs

A large part of the human genome encodes RNAs, which are not translated into proteins (e.g. RNAs with regulatory functions, etc.) and which can be polyadenylated or not [21]. Non-coding RNAs can be arbitrarily subdivided in two categories: small non-coding RNAs (<200 nucleotides) and long non-coding RNAs (lncRNAs). The investigation of small non-coding RNAs, including mature micro RNA (miRNA), piRNA, small nuclear RNA (snRNA) and some small nucleolar RNA (snoRNA) is limited with the preparations method used herein, and more adequate protocols are available for this task [22, 23]. The library construction methods used herein include size selection steps, where smaller fragments are removed (typically <100 bp with AMPure XP beads from Beckman Coulter). Therefore, we focused our comparative analysis of the different protocols on the lncRNAs. The number of newly characterized lncRNAs is growing rapidly and we used herein the lncRNA reference database annotated and curated by the Gencode project group (Genecode 18, see Methods), which is embedded in Ensembl (v70). Following the Gencode definition, lncRNAs were classified into seven subtypes and are currently totalling 13,238 lncRNAs consisting mostly of natural antisense transcripts, intergenic and intronic lncRNAs (Table 2). In HEK293 cells, we detected 1,946 and 2,112 lncRNAs in poly(A)+ RNA-seq derived from Qiagen and TRIzol-extracted RNAs, respectively (rpkm > =0.5). A similar number of lncRNAs were detected within the RiboZero RNA-seq from Qiagen and TRIzol RNAs (1,885 and 2,174 respectively). From a total of 2,643 unique lncRNAs detected when combining data from all protocols, 1,536 were found by all four methods, 324 were unique to poly(A)+ RNA-seq and 447 were unique to RiboZero RNA-seq (Additional file 1: Figure S7). We observed that additional lncRNAs were detected in TRIzol RNA over Qiagen RNA samples (8.5% and 15% for poly(A)+ RNA-seq and and RiboZero methods, respectively) (Table 2). Interestingly, 324 lncRNAs were detected only by poly(A)+ RNA-seq, of which 208 were antisense transcripts (Additional file 1: Figure S7, Additional file 4: Table S3). Conversely, 447 were found exclusively in RiboZero RNA defining either intronic sense RNAs (182) or large intervening non-coding RNAs (lincRNAs) (114) classes (Additional file 4: Table S3).

The overall distribution of lncRNA expression levels was similar across methods (median = 1 rpkm) and was lower than for protein coding genes (median = 8.2 rpkm) in all methods, confirming previous results (Additional file 1: Figure S8) [24]. However, three lncRNAs (RN7SL1, RPPH1, SNORD3A) displayed dramatically high expression values in all RiboZero protocols (Additional file 4: Table S3). The total number of reads falling into this RNAs accounted for ~65% of all lncRNA read sequences in both RiboZero RNA-seq total RNA datasets. Such highly expressed entities are problematic in RNA-seq datasets as it considerably lowers the sequencing depth of the other RNAs. The RN7SL1 RNA molecule is part of the signal recognition particle (SRP) complex, which mediates co-translational insertion of secretory proteins into the lumen of the endoplasmic reticulum and is partially homologous to Alu DNA [25]. The ribonuclease P RNA component H1 (RPPH1) is a known poly(A)- RNA component of the RNase P ribonucleoprotein [26]. SNORD3A is a known abundant snoRNA involved in the processing of rRNA precursors [27]. These RNAs are expressed at levels that are over 1,200 fold higher in RiboZero RNA-seq and thus are predominantly non-polyadenylated. These three highly abundant lncRNAs could be specifically depleted by adding corresponding specific probes in the RiboZero protocol, thus improving the sequencing depth of the remaining RNAs.

Gathering information on the non-polyadenylated fraction of the RNA is the most attractive advantage of using RiboZero versus poly(A)+ selection. However, in the bulk of transcripts obtained from RiboZero data, it remains unclear how to differentiate between transcripts that are polyadenylated, not polyadenylated and/or bimorphic [7]. Under the simple assumption that non-polyadenylated transcripts would be found at higher levels in RiboZero data, we applied following arbitrary filters: RPKM <0 .5 in poly(A)+ and > =1 in RiboZero. We found only 94 lncRNAs that passed these criteria (Additional file 4: Table S3). A fraction of lncRNAs were expressed at higher levels in RiboZero samples, although they were also detected in poly(A)+ samples (e.g. RN7SL1), suggesting the coexistence of two forms of those transcripts (poly(A)+ and poly(A)-).

**Figure 6:**
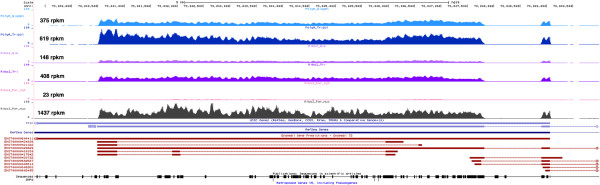
**Snapshot of the XIST lncRNA.** Representation of the normalized read coverage of XIST for each method in the UCSC browser.

Finally, lncRNAs expressed from intergenic regions of the genome (lincRNAs) have been the focus of increasing attention in the last years as they are emerging as key regulators of diverse cellular processes and several thousands have been described in human and mouse [28–31]. The proportion of lincRNAs that was detected herein was relatively low and similar across methods, except for nuclear RNA, with an average of detection close to 9% of the 6,453 annotated lincRNAs to date (Table 2). Actually, more lincRNAs were found in poly(A)+ selected RNA samples than in all other protocols (Table 2). This reflects the fact that nearly all lincRNAs have a mRNA similar structure as they are capped, spliced and polyadenylated, although they do not encode proteins [32, 33]. Further, many are retained primarily in the nucleus as corroborated by the fact that we detected nearly twice as many lncRNAs in the nucleus than in the cytoplasm (Table 2). The X-inactive specific transcript (XIST) represented an illustrative example of the differences between RNA extraction methods in releasing the RNA content of the nucleus. XIST is one of the first identified and best-studied lincRNAs. It is capped, spliced and polyadenylated and accumulates mainly in the nucleus [34–36]. Its expression pattern clearly reflected the expected localization, with lower expression in the cytoplasm (23 rpkm) in comparison to the nucleus (1437 rpkm). However, we noticed systematically higher expression levels of XIST in TRIzol derived RNA than in Qiagen extracted RNA (1.6-2.8 fold higher) (Figure 6).

### Impact on splicing analysis

RNA-seq can be instrumental for detecting alternative splicing events [37, 38], and most algorithms designed for detecting ASEs rely on statistical tests exploiting the information from sequence reads localized at exon-exon junctions. However, this approach requires relatively high sequencing depth of the transcripts in order to be able to detect most of the junctions. To estimate how many junction reads were detected by the different methods, we mapped all sequence read using the spliced aligner TopHat 2 [39] (see Methods). The fraction of junction reads were the highest, for Qiagen RNA/poly(A)+ RNA-seq and cytoplasmic RNA/RiboZero RNA-seq representing ~10,5%, of the mapped reads, (Additional file 5: Table S4). TRIzol RNA/poly(A)+ RNA-seq and Qiagen RNA/RiboZero RNA-seq performed equally well (~9,3%). However, the combination TRIzol RNA/RiboZero RNA-seq underperformed with only 6,4% of the reads mapping to splice junctions. As expected, only 2,9% of sequenced nuclear RNA corresponded to junction reads, in line with the fact that mature transcripts are exported into the cytosol. In summary, Qiagen RNA/poly(A)+ RNA-seq performed best for the number of spliced identified junctions, but were overall comparable to the TRIzol RNA/poly(A)+ RNA-seq and Qiagen RNA/RiboZero outcomes (Additional file 5: Table S4), in line with previous data comparing Ribominus and poly(A)+ selected RNA-seq [16].

In summary, the procedure of choice for identifying alternative splicing events by NGS remains the poly(A)+ RNA-seq strategy (in combination with the Qiagen RNA preparation containing less nuclear material), whereas the analysis is more challenging with RiboZero sequencing, for which the sequencing depth should also be increased to compensate for the large fraction of reads localized outside of coding regions.

## Discussion

The majority of NGS-based transcriptome analysis had interrogated the polyadenylated fraction of RNAs extracted from tissues or whole cells, assuming that most known mature mRNAs are polyadenylated and located in the cytoplasm [40, 41]. Non-coding RNAs can also be polyadenylated and are captured along with mRNA using oligo-d(T) tagged beads. In principle,,mRNA quantification through poly(A)+ RNA-seq is thought to be reliable and accurate whereby the contribution of nuclear RNA to the total RNA population has been considered negligible for the study of mature coding transcripts [42]. The recent introduction of ribosomal RNA depletion protocols in the NGS procedure enabled to extend the view of the transcriptome to the poly(A)- fraction of the RNA, and this technique becomes increasingly popular, also due to its low requirements in terms of total RNA material.

Starting from the same source of HEK293 cells, we compared two commonly used RNA extractions protocols (Qiagen and TRIzol) coupled with two RNA-seq approaches (poly(A)+ and RiboZero) and described the qualitative and quantitative differences in the respective data output. In turn, the fraction of reads mapping to intronic regions was higher in RNA extracted with TRIzol than in RNA extracted with a Qiagen protocol, and was mostly attributed to hnRNA localized in the nucleus. These differences were particularly pronounced after using rRNA depleted total RNA sequencing, while being less dramatic with poly(A)+ RNA-seq. The relative differences observed in the proportion of exonic, intronic and intergenic sequences were protocol dependent. These observations argue for processing of RNA in a highly controlled and standardized manner. For example, cells or tissue should not be stored in varying volumes of e.g. TRIzol for varying periods of time, which will consequently release variable amounts of nuclear material, introducing differences in the exonic/intronic read distribution. Whenever possible, it is preferable to keep the samples as fresh frozen material (in liquid nitrogen), and perform the nucleic acid extraction in one step.

The expression levels of protein coding genes quantified in rpkm tended to be lower in RiboZero RNA-seq than in poly(A)+ RNA-seq, resulting in a reduced sequencing depth in exonic regions when the number of reads in intronic sequences was high (e.g. Trizol extractions). This issue might need particular attention when conducting metadata analysis exploiting RNA-seq data generated by different extraction and selection protocols, especially if the expected expression changes between samples are of small amplitude (e.g. trisomic versus disomic chromosome configurations in aneuploidies).

## Conclusions

Previous studies have reported an impact of the presence of nuclear RNA on steady state mRNA expression analysis [5, 43]. However, to the best of our knowledge, no report has, as yet, highlighted the influence of the extraction method on the released amount of nuclear RNA. Gathering information on non-polyadenylated transcripts is the main advantage of RiboZero over poly(A)+ RNA-seq. It was reported that the fraction of non-poly (A) or bimorphic transcripts could be two times larger than the poly(A)+ RNAs in the cytoplasm of HeLa cells cells [40, 44]. However, Yang et al. found that the majority of transcripts were polyadenylated in HeLA and hESC H9 cells [7]. In the bulk of transcripts identified in the RiboZero procedure, it remains difficult to differentiate *a priori* between polyadenylated and not polyadenylated, or bimorphic transcripts. The gain of information resulting from RiboZero RNA-seq might be dimmed, if one cannot discriminate between the RNA sub-populations, if expression data are biased and if the power of detecting alternative splicing is reduced. It is therefore advisable to benchmark the RiboZero method with a poly(A)+ selection, when high resolution analysis of the transcriptome is required. It is possible to prepare and index these two fractions sequentially from the same source of starting material, with the advantage of capturing both polyadenylated and non-polyadenylated fractions and to finally sequence those in one experiment.

## Methods

### Cell culture

The commercially available HEK 293 T cell line was purchased from ATCC**®** (#CRL-11268). The cells were grown in parallel in 75 cm^2^ flasks (37°C, 5% CO_2_) for 3 days from a P16 passage in D-MEM medium (GIBCO #31885-049) supplemented with 1% Penicillin/Streptomycin (GIBCO #15140-122) and 5-10% FCS (Sigma #7524). After two passages all cells were pooled together and washed with two times with PBS and splitted into aliquots of 1 million cells. Cell pellets were frozen in liquid nitrogen and further used for RNA extraction.

### TRIzol total RNA Extraction (RNA1, 2)

Frozen cell pellets were re-suspended in 500 μl TRIzol (Life Technologies #15596-018), briefly vortexed and 200 μl Chroroform (Merck #102445) was added. Heavy MaXtrack Tubes (Qiagen #1038988) were used the phase separation. The RNA precipitation was done with 10 μg RNase free Glycogen and 500 μl Isopropanol (Merck #109634). The RNA pellets were washed with 1 ml of 70% ice cold Ethanol.

### Qiagen total RNA extraction (RNA3, 4)

Total RNA from cell pellets was purified using the RNeasy Mini Kit (Qiagen, #74104) and frozen cells were re-suspended in 350 μl of buffer RLT and the lysates were passed 5 times through a 20-gauge needle, and processed following the manufacturer’s instructions.

### Paris cytoplasmic (RNA5, 6) and nuclear (RNA7, 8) RNA extraction

PARIS**™** Kit (Life Technologies, #AM1921) was used to separately isolate nuclear and cytoplasmic RNA from actively grown cells, following the manufacturer instructions.

### DNase treatment

All RNAs were DNase treated using TURBO DNA-*free*™ (Life Technologies, #AM1907) following the manufacturer instructions. In brief, digestions were performed at 37°C for 20 minutes and using 2U of TURBO DNase in 90 μl reaction. Ethanol precipitation was done with 3 M NaOAc and 10 μg Glycogen. All concentrations were evaluated with the Qubit 2.0 Fluorometer (Invitrogen) and RNA quality was monitored by Bioanalyzer.

### poly(A)+ strand-specific RNA libraries

poly(A)+ selected RNA libraries were prepared following a protocol published recently and preserving the strand information [45]. The starting amount of total RNA was 500 ng.

### Ribosomal RNA depleted strand-specific RNA libraries

rRNA was removed from 200 ng of total RNA using the RiboZero^TM^ Magnetic Gold kit (Epicentre, #MRZG12324) following the manufacturer instructions. The rRNA depleted RNA pellets obtained after the ethanol precipitation step was re-suspended in 0.5 μl of RNase free water. The samples were than further processed as described in [45], but starting at “Make RFP” (step 13, p.81) of the Illumina TruSeq RNA Sample Preparation v2 (HT) protocol (Part#15026495Rev.A) and with minor modifications in the “Make CDP” part (p.84): steps 1 and 2 were omitted; at step 3 the whole reaction (20 μl) was used; at step 6, 9 μl of the Superscript II and First Strand Master mix was added instead of 8 μl.

### Sequencing and mapping

Sequencing was carried out on the HiSeq2500 with 2 × 51 cycles and using version 3 of the Illumina sequencing chemistry. All reads were aligned to the NCBI37/hg19 assembly of the human reference genome using BWA (v.0.5.9-r16) [46]. Only read with MAPQ ≥ 1 were considered for expression analysis.

### Data analysis

Coordinates for protein coding genes and corresponding exons were downloaded from the Ensembl Genes (v70) database using BioMart tool and Homo sapiens genes (GRCh37.p3) dataset. Only the intervals on chromosomes 1–22, X, Y, M with merged overlapping exons belonging to the same gene were used to calculate exons hits. Non-overlaping introns and intergenic regions were generated using BEDTools [47] and UCSC hg19 chromosomes sizes. A custom script was used to count the number of sense- and antisense reads overlap each exon interval. To count introns and intergenic hits, only reads that are completely inside of an intronic or intergenic interval were used. The strand-specific RNA-seq protocols used enable including only reads belonging to the original orientation of transcription orientation for the calculation of exon and intron. Exons and intron RPKM values were calculated according to exon and intron hits, respectively and normalized against the library size (total MAPQ ≥ 1 reads) and to the merged length of the coding sequence (or intronic sequence for intron RPKM) of each gene.

The analysis of long non-coding RNAs was based on Ensembl (v70), which is based on the annotation and curation within the Gencode project (http://www.gencodegenes.org/). The definition and classification of each lncRNA subtype can be found at http://www.gencodegenes.org/gencode_biotypes.html. RPKM values were calculated as described above.

The generalized Logarithm (GLog) of each rpkm expression value (e.g. for scatter plots) was calculated using following formula: , where *x* is the rpkm value. Plots and tables were generated in R and/or with Microsoft excel 2011.

### Differential expression

The analysis of differential expression Analysis was conducted with the Bioconductor Package NOIseq, Version 1.3.0 [17]. The input for NOISeq analysis were given as gene read count, which were normalized by the total number of read counts in annotated regions, excluding intronic and intergenic reads. This comprises a set of 50,800 annotated RNAs from Ensembl (v62). As a cut off, we considered protein coding genes to be dysregulated when the probability was higher than 80% (q > =0.8).

### Spliced alignments

The paired reads were aligned with tophat 2.0.3 using Bowtie 1 and junction coordinates based on Ensembl (v62). The resulting spliced alignments were used to count reads that span known junctions with a 5 base seed on the donor and acceptor side. The junctions were defined by unique genome position.

### Availability of supporting data

The sequence data set supporting the results of this article are available in the European Nucleotide Archive under the accession number PRJEB4197 (https://www.ebi.ac.uk/ena).

## Electronic supplementary material

Additional file 1: **Supplementary data file.** The supplementary data files contains the supplementary figures and legends S1-S8. (PDF 2 MB)

Additional file 2: Table S1: Mean exonic and intronic RPKM values of protein coding genes. The cumulated length of the exonic and intronic sequence used for the rpkm calculation is given in columns 7 and 8, respectively. The standard deviation (SD) of the is shown in the columns next to each mean rpkm values.The gene annotation is based on the Ensembl (v70). (XLS 8 MB)

Additional file 3: Table S2: List of differentially expressed protein coding genes (1). The table lists each gene detected as differentially expressed by the NOISeq algorithm (see Material and Methods), for each pairwise comparison listed in the first column. The gene annotation used as input is based on the Ensembl (v62). The values given in the tables correspond to the NOISeq output (M and D values, probability (q > 0.8), ranking) and are described at: http://www.bioconductor.org/packages/2.13/bioc/vignettes/NOISeq/inst/doc/NOISeq.pdf. (ZIP 5 MB)

Additional file 4: Table S3: Mean RPKM expression of lncRNAs. The table lists for each annotated lncRNA it’s expression values across the different methods. The lncRNA annotation is based on the Ensembl version 70. (XLSX 1 MB)

Additional file 5: Table S4: Splice Junctions statistics. The table lists mapping statistics of exon junction reads of protein coding genes derived from the TopHat alignment. (XLSX 58 KB)

## Abbreviations

rRNA: ribosomal RNA
mRNA: messenger RNA
NGS: Next generation sequencing
RNA-seq: RNA sequencing
hnRNA: Precursor or unprocessed mRNA
lnc RNA: Long non coding RNA
lincRNA: Long intergenic non coding RNA
snRNA: Small nuclear RNA
snoRNA: Small nucleolar RNA
miRNA: MicroRNA
piRNA: Piwi-interacting RNA
rpkm: Reads per kilobase per million.
