# Supplementary material for: Influence of RNA extraction methods and library selection schemes on RNA-seq data
Source: BMC Genomics. 2014 Aug 11;15(1):675. doi: 10.1186/1471-2164-15-675 (PMC4148917; doi:10.1186/1471-2164-15-675)
Supplement: Supplementary file 1 — Additional file 1: Supplementary data file. The supplementary data files contains the supplementary figures and legends S1-S8. (PDF 2 MB) [file 12864_2014_6375_MOESM1_ESM.pdf]

SUPPLEMENTARY DATA

Influence of RNA extraction methods and library selection schemes on RNA-seq data

Marc Sultan[1], Vyacheslav Amstislavskiy[1], Thomas Risch[1], Moritz Schuette[1], Simon Dökel[1], Meryem Ralser[1], Daniela Balzereit[1], Hans Lehrach[1], Marie-Laure Yaspo[1].

Supplementary Tables legends

Table S1: **Mean exonic and intronic RPKM values of protein coding genes**. The cumulated length of the exonic and intronic sequence used for the rpkm calculation is given in columns 7 and 8, respectively. The standard deviation (SD) of the is shown in the columns next to each mean rpkm values.The gene annotation is based on the Ensembl (v70).

Table S2: **List of differentially expressed protein coding genes (1)**. The table lists each gene detected as differentially expressed by the NOISeq algorithm (see Material and Methods), for each pairwise comparison listed in the first column. The gene annotation used as input is based on the Ensembl (v62). The values given in the tables correspond to the NOISeq output (M and D values, probability (q>0.8), ranking) and are described at:
http://www.bioconductor.org/packages/2.13/bioc/vignettes/NOISeq/inst/doc/NOISeq.pdf.

Table S3: **Mean RPKM expression of lncRNAs**. The table lists for each annotated lncRNA it's expression values across the different methods. The lncRNA annotation is based on the Ensembl version 70.

Table S4: **Splice Junctions statistics**. The table lists mapping statistics of exon junction reads of protein coding genes derived from the TopHat alignment.

Supplementary Figures S1-S8

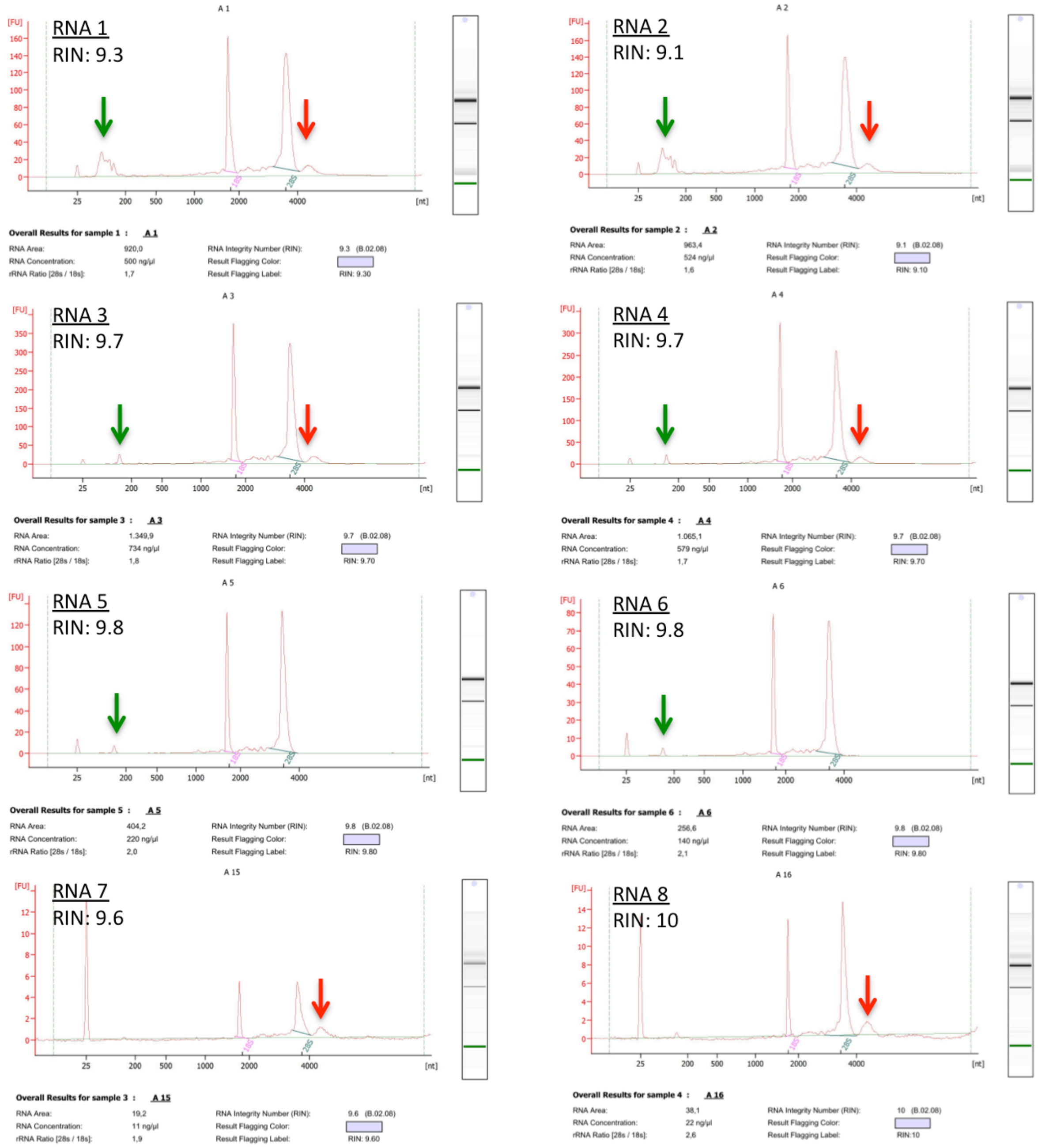

<u>**Figure S1**</u>: **Bioanalyser Profiles.** The green arrows point to small RNA peaks (<200 ntdes andt red arrows to long RNAs (>4,000 ntdes).

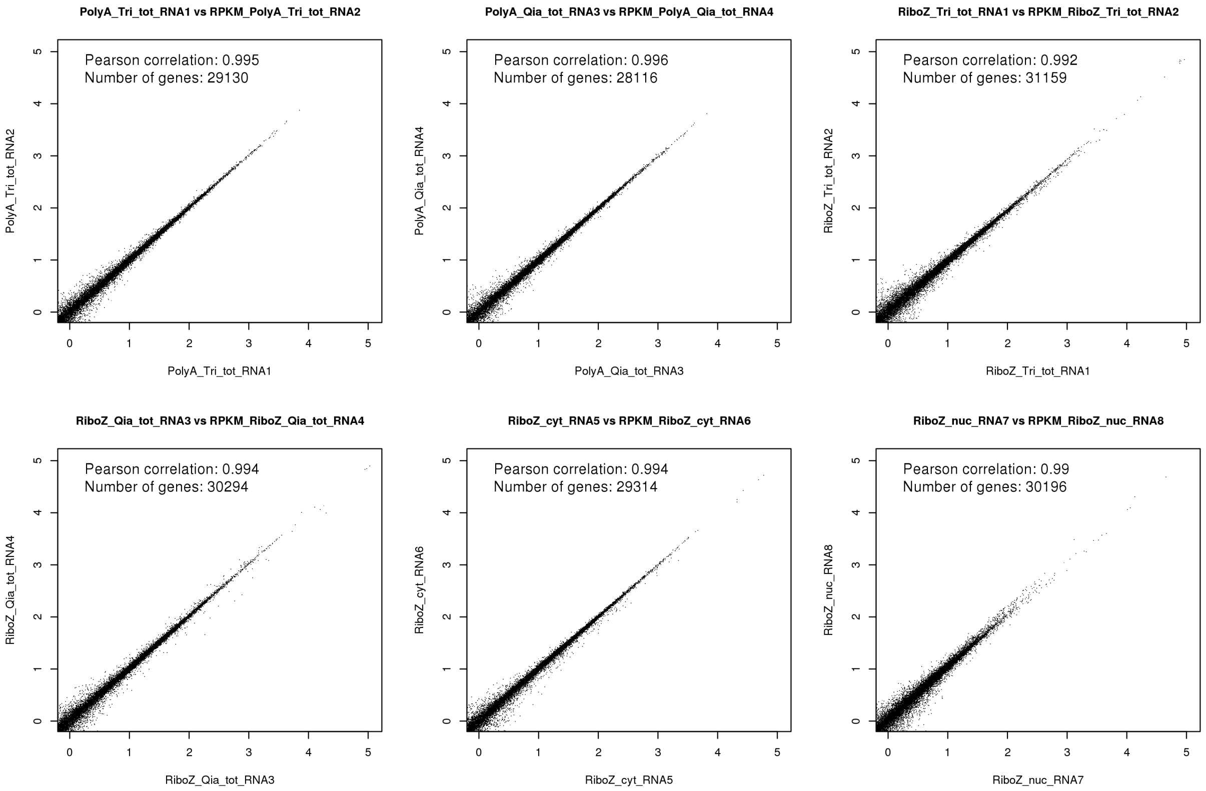

Figure S2: **Scatter plots of replicates**. The generalized Log of rpkm values were plotted against each other between the 2 replicates of a given experiment (see methods). Null values in both replicates were removed and the number of genes kept is shown on each plot along with Pearson's correlation coefficient.

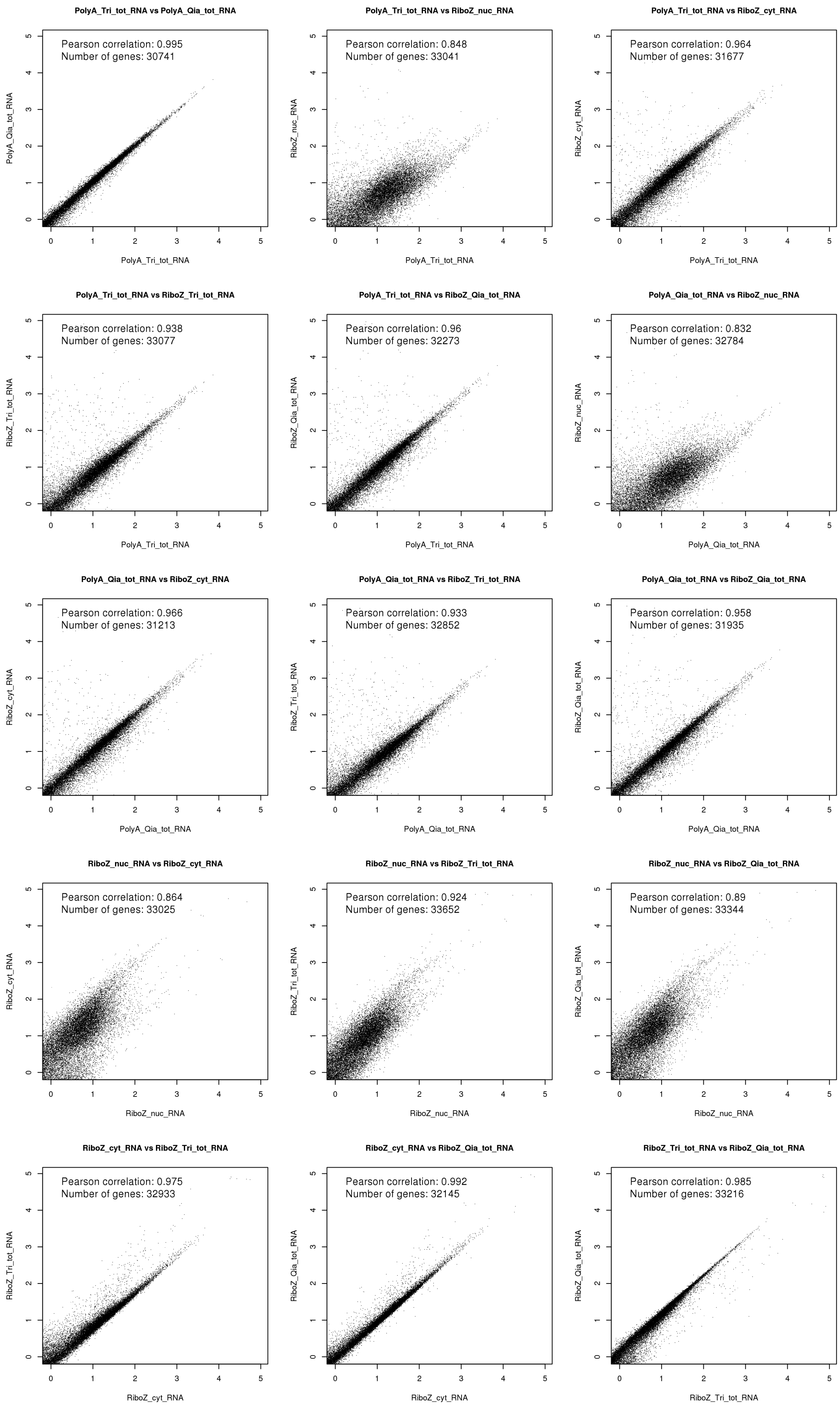
PolyA_Tri_tot_RNA vs PolyA_Qia_tot_RNA
Pearson correlation: 0.995
Number of genes: 30741
PolyA_Tri_tot_RNA
PolyA_Qia_tot_RNA
PolyA_Tri_tot_RNA vs RiboZ_nuc_RNA
Pearson correlation: 0.848
Number of genes: 33041
PolyA_Tri_tot_RNA
RiboZ_nuc_RNA
PolyA_Tri_tot_RNA vs RiboZ_cyt_RNA
Pearson correlation: 0.964
Number of genes: 31677
PolyA_Tri_tot_RNA
RiboZ_cyt_RNA
PolyA_Tri_tot_RNA vs RiboZ_Tri_tot_RNA
Pearson correlation: 0.938
Number of genes: 33077
PolyA_Tri_tot_RNA
RiboZ_Tri_tot_RNA
PolyA_Tri_tot_RNA vs RiboZ_Qia_tot_RNA
Pearson correlation: 0.96
Number of genes: 32273
PolyA_Tri_tot_RNA
RiboZ_Qia_tot_RNA
PolyA_Qia_tot_RNA vs RiboZ_nuc_RNA
Pearson correlation: 0.832
Number of genes: 32784
PolyA_Qia_tot_RNA
RiboZ_nuc_RNA
PolyA_Qia_tot_RNA vs RiboZ_cyt_RNA
Pearson correlation: 0.966
Number of genes: 31213
PolyA_Qia_tot_RNA
RiboZ_cyt_RNA
PolyA_Qia_tot_RNA vs RiboZ_Tri_tot_RNA
Pearson correlation: 0.933
Number of genes: 32852
PolyA_Qia_tot_RNA
RiboZ_Tri_tot_RNA
PolyA_Qia_tot_RNA vs RiboZ_Qia_tot_RNA
Pearson correlation: 0.958
Number of genes: 31935
PolyA_Qia_tot_RNA
RiboZ_Qia_tot_RNA
RiboZ_nuc_RNA vs RiboZ_cyt_RNA
Pearson correlation: 0.864
Number of genes: 33025
RiboZ_nuc_RNA
RiboZ_cyt_RNA
RiboZ_nuc_RNA vs RiboZ_Tri_tot_RNA
Pearson correlation: 0.924
Number of genes: 33652
RiboZ_nuc_RNA
RiboZ_Tri_tot_RNA
RiboZ_nuc_RNA vs RiboZ_Qia_tot_RNA
Pearson correlation: 0.89
Number of genes: 33344
RiboZ_nuc_RNA
RiboZ_Qia_tot_RNA
RiboZ_cyt_RNA vs RiboZ_Tri_tot_RNA
Pearson correlation: 0.975
Number of genes: 32933
RiboZ_cyt_RNA
RiboZ_Tri_tot_RNA
RiboZ_cyt_RNA vs RiboZ_Qia_tot_RNA
Pearson correlation: 0.992
Number of genes: 32145
RiboZ_cyt_RNA
RiboZ_Qia_tot_RNA
RiboZ_Tri_tot_RNA vs RiboZ_Qia_tot_RNA
Pearson correlation: 0.985
Number of genes: 33216
RiboZ_Tri_tot_RNA
RiboZ_Qia_tot_RNA

<u>Figure S3</u>: **Scatter plots of replicates**. The generalized Log of mean rpkm values of 2 replicates were plotted against each other between the 2 different experiments (see methods). Null values in both replicates were removed and the number of genes kept is shown on each plot along with Pearson's correlation coefficient.

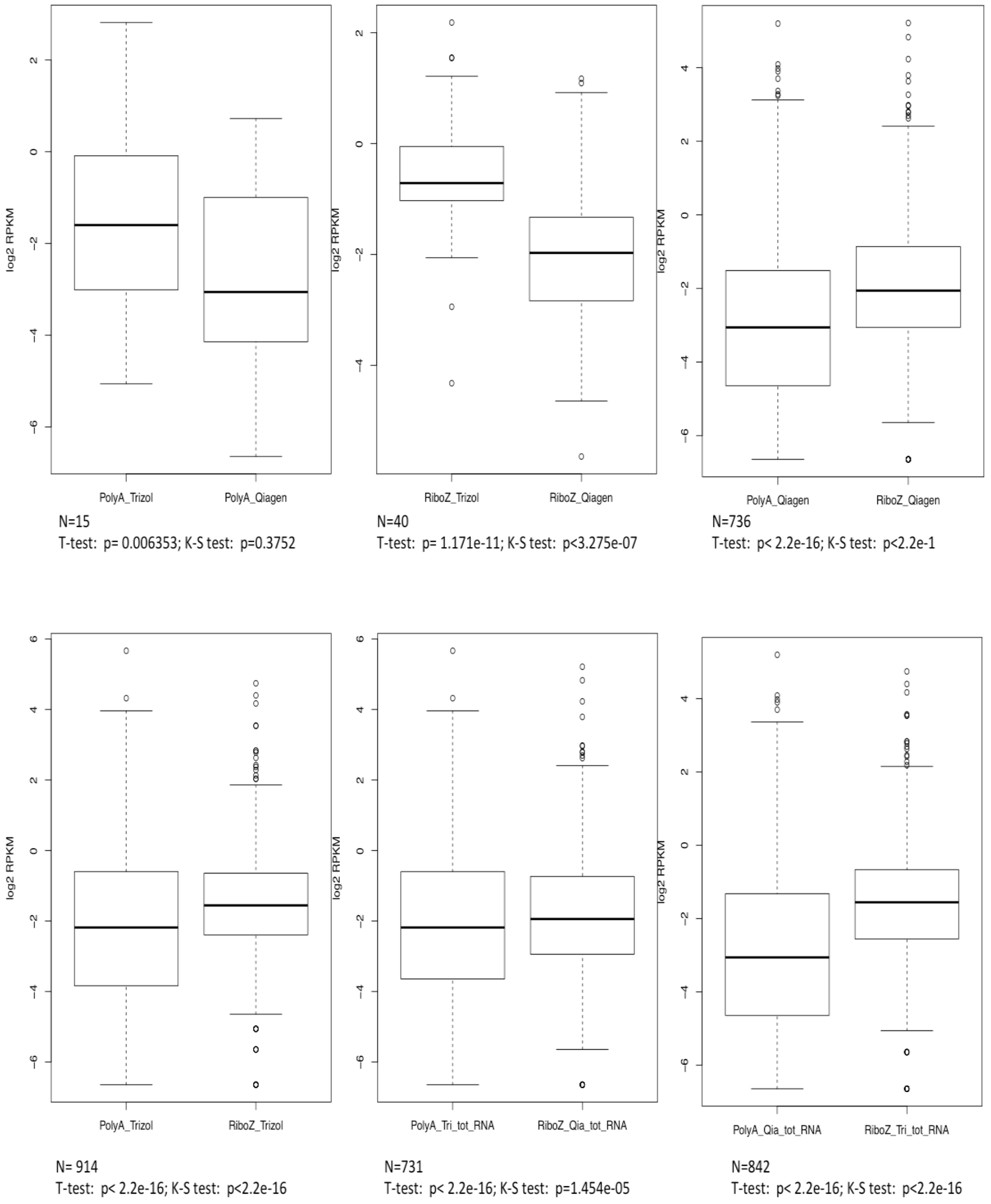

<u>Figure S4</u>: **Distribution of intronic expression levels of differentially expressed protein coding genes**. The boxplots show the distribution of the intronic rpkm values of genes identified as differentially expressed between two methods (see methods). The number of genes taken into account for each pairwise comparison is given below each plot (N). This number can be lower thatn the total number of differentially expressed protein coding genes as not all do possess introns. The Welch T-test (T-Test) and the Kolgomorov-Smirnov test (K-S test) were applied to test whether two distribution are significantly different. The respective p values are given below each plot.

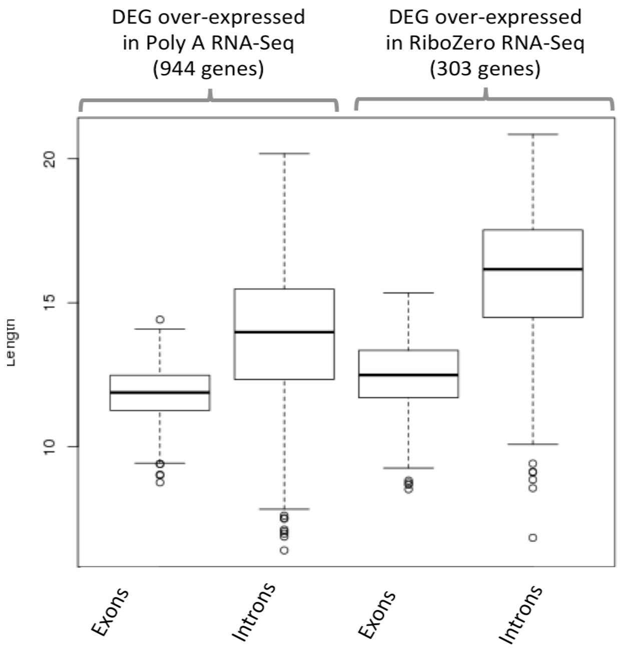

Figure S5: **Distribution of exonic and intronic gene length of differentially expressed protein coding genes.** The boxplots show the distribution of the length of introns and exons of the differentially expressed protein coding genes (DEG) that were identified by comparing Poly(A)+ and RiboZero RNA sequencing protocols. Genes with higher expression in Poly(A)+ RNA data had a longer intronic (median= 73,4 kb; $p_{Kolmogorov\text{-}Smirnov}$<2.2e-16) and exonic (median=5.8 kb, $p_{Kolmogorov\text{-}Smirnov}$=2.4e-14) median sequence size than genes with higher expression in RiboZero RNA-seq data ($median_{intron}$ = 16,1 kb; $median_{exon}$= 3,8 kb).

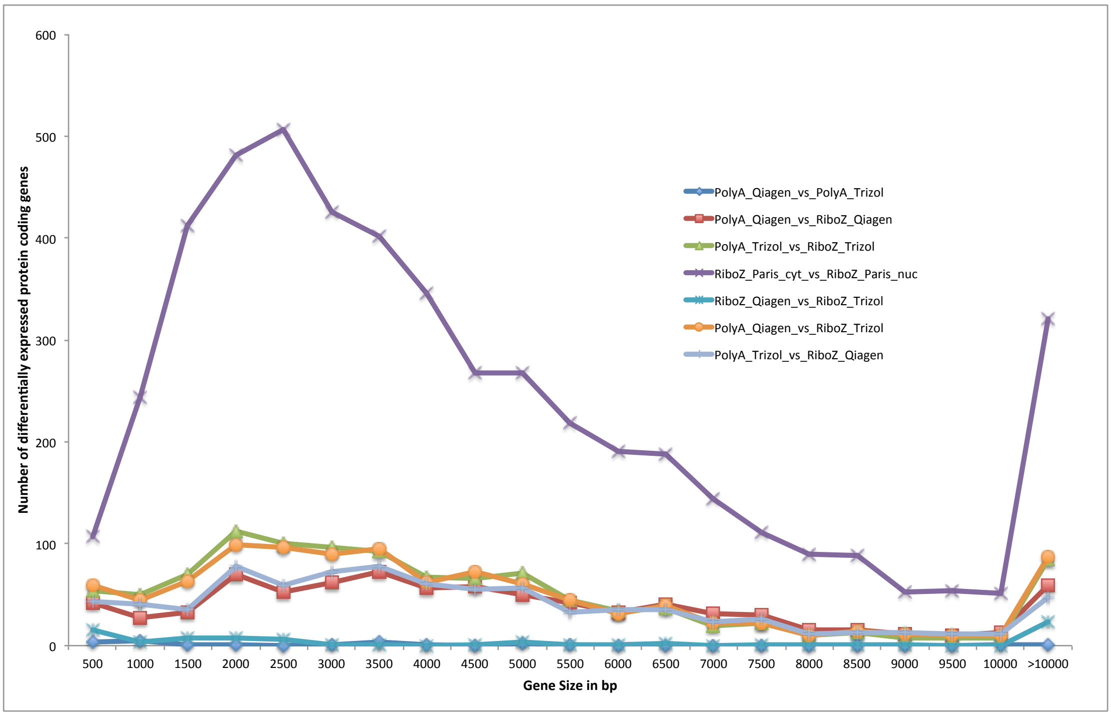

Figure S6: **Size Distribution of differentially expressed protein coding genes.** Differentially expressed genes in each pairwise comparison were divided in 500 bp bins according to the size of their coding sequence (x-axis). The number of identified dysregulated genes in each bin was plotted on the y-axis.

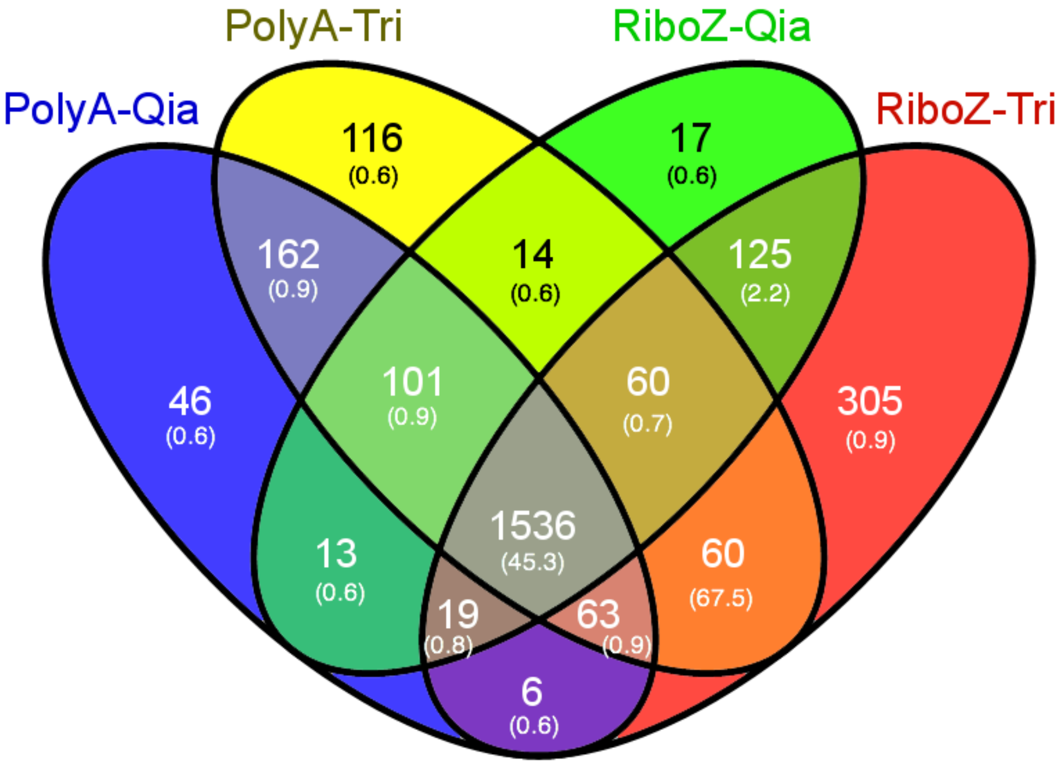

Figure S7: **Venn Diagram of lncRNAs**. The diagram shows the number of commonly detected lncRNA (>=0.5 rpkm) between the different protocols. For each intersection, the number of detected genes is given as well as the average rpkm value (in brackets).

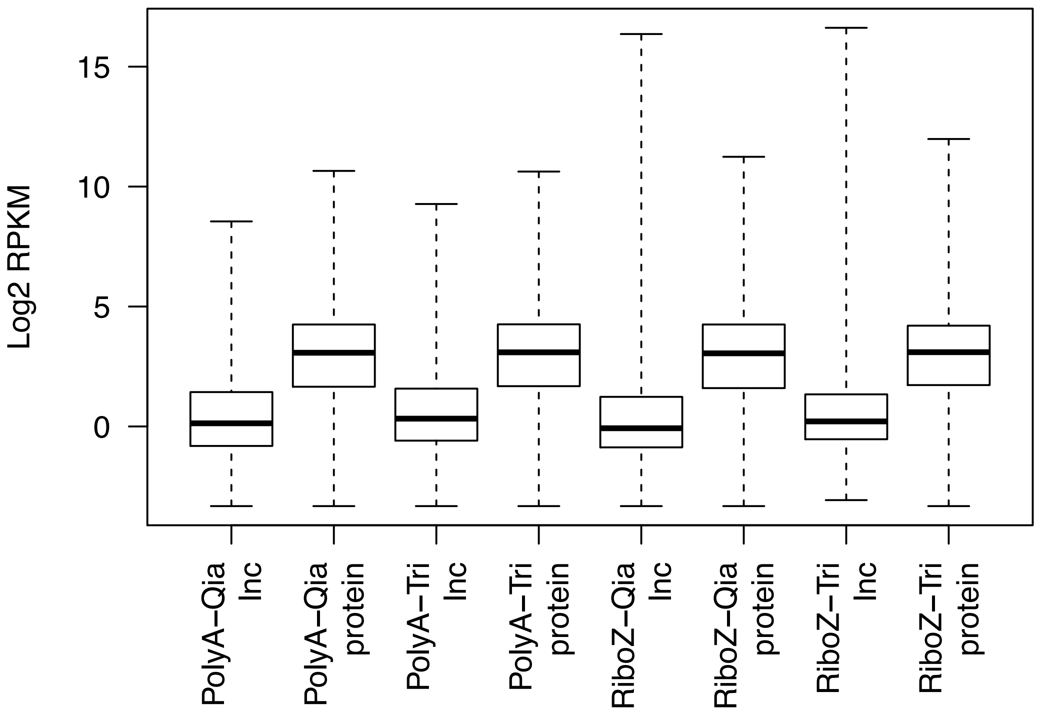

Figure S8: Boxplots of the rpkm distributions in protein coding genes and lncRNAs for each experimental set
